# Effect of elastic oral appliance chewing on frontal lobe activity

**DOI:** 10.1002/cre2.710

**Published:** 2023-01-10

**Authors:** Yuu Yamamoto, Masahiro Ryu, Takayuki Ueda, Yoshinori Sasaki, Kaoru Sakurai

**Affiliations:** ^1^ Department of Removable Prosthodontics and Gerodontology Tokyo Dental College Tokyo Japan

**Keywords:** frontal lobe, mastication, near‐infrared, spectroscopy

## Abstract

**Objectives:**

Chewing increases frontal lobe activity, resulting in improved memory, learning ability, and response reaction time. This study aimed to assess the effects of elastic oral appliance chewing on the activities and functions of the frontal lobe.

**Methods:**

The study participants were 15 healthy men with full dentulous (mean age, 27.4 ± 4.1 years). A prospective crossover design was used to assess frontal lobe activities and functions. Changes in frontal lobe activities were measured with near‐infrared spectroscopy (NIRS). At baseline, the participants were assessed in the resting state. Changes in channels #7, representing right frontal lobe activities by NIRS, and #10, representing left frontal lobe activities, during the first and second chewing periods in a total of two periods were evaluated. Frontal lobe functions were measured using the Trail Making Test Part A (TMT‐A) in the resting state and after elastic oral appliance or gum chewing. These values were compared with each period.

**Results:**

Elastic oral appliance chewing caused significant differences between the baseline and first chewing periods for channel #7 (*p* = .032) and significant differences between the baseline and second chewing periods for channels #7 and #10 (*p* < .001 and *p* < .001, respectively) using NIRS. Moreover, significant differences were found in the TMT‐A results between the resting state and elastic oral appliance chewing (*p* = .04).

**Conclusions:**

Elastic oral appliance chewing improves frontal lobe activities to a level similar to that obtained with gum chewing.

## 1 BACKGROUND

The population with dementia is increasing globally (Prince et al., 2015). Approximately 10% of mild dementia cases progress to severe dementia annually (Bruscoli & Lovestone, 2004). Mild cognitive impairment is a predementia condition, and a further decline in cognitive functions can progress to dementia. However, no treatment has been established for this condition. Thus, cognitive function decline should be prevented, (Livingston et al., 2017), and a training method that can increase brain activities must be used to prevent such a decline.

Cognitive functions are associated with oral motor functions (Kugimiya et al., 2019; Watanabe et al., 2018). Several studies have reported the effects of chewing on brain functions, including its influence on autonomic and central nervous system activities and cognitive functions. According to Ohta et al. (2017, 2018), chewing enhances the autonomic nervous system activities of individuals aged ≥70 years. Moreover, gum chewing increases the activities in the inferior frontal gyrus, which is located in the frontal lobe (Hirano et al., 2013; Quintero et al., 2013). These studies have indicated that gum chewing affects frontal lobe activities.

Furthermore, gum chewing affects cognitive functions, working memory, and learning ability. The prefrontal cortex (PFC) is involved in working memory, (Kane & Engle, 2002), and PFC damage causes cognitive impairment in schizophrenia (Maas et al., 2017). Mastication exercise using chewing gum was also found to improve working memory and learning ability (Fukushima‐Nakayama et al., 2017; Jiang et al., 2015) and activate the parasympathetic nervous system (Ohta et al., 2017, 2018). Moreover, this exercise can improve reaction time in response to instructions, wakefulness, and cognitive functions (Hirano et al., 2013; Sakamoto et al., 2009).

Previous studies have shown that chewing increases frontal lobe activities, resulting in improved memory, learning ability, and reaction time in response to stimulation. Moreover, chewing improves cognitive functions, including the ability to execute visual and external tasks.

Chewing gums and other foods have been used in studies that assessed the effects of chewing on brain activities, including those of the frontal lobe (Kamiya et al., 2010; Ohta et al., 2018; Shoi et al., 2014). However, wearers of removable dentures find it difficult to chew gum because it is likely to adhere to denture base materials (Wada et al., 2016). In chewing exercises, the use of food instead of chewing gum is disadvantageous because of the shorter exercise duration. Thus, we considered that chewing an elastic oral appliance made from soft acrylic resin that covers the mandibular dental arch can increase frontal lobe activities. By using this elastic oral appliance, chewing exercises can be performed without chewing food or gum.

This study aimed to determine the effect of the elastic oral appliance chewing exercise on frontal lobe activities and functions. Accordingly, we hypothesized that the elastic oral appliance chewing exercise could increase frontal lobe activities.

## 2 METHODS

### 2.1 Participants

The study participants were 15 healthy men with full dentulous (mean age, 27.4 ± 4.1 years). Those with a previous medical history of cerebrovascular disorders or cranial nerve diseases were excluded. Moreover, only male participants were included to eliminate sex‐associated differences in brain reactivity to external stimuli (Gao et al., 2018; Okada et al., 1993).

After explaining the objective and methods of the study, written informed consent was obtained from the participants. This study was performed after obtaining approval (No. 754) from the Ethics Committee of Tokyo Dental College. The study was fully designed and performed in accordance with relevant guidelines and regulations, particularly those set out in the Declaration of Helsinki pertaining to the ethical treatment of human subjects.

### 2.2 Elastic oral appliance

**Figure 1:**
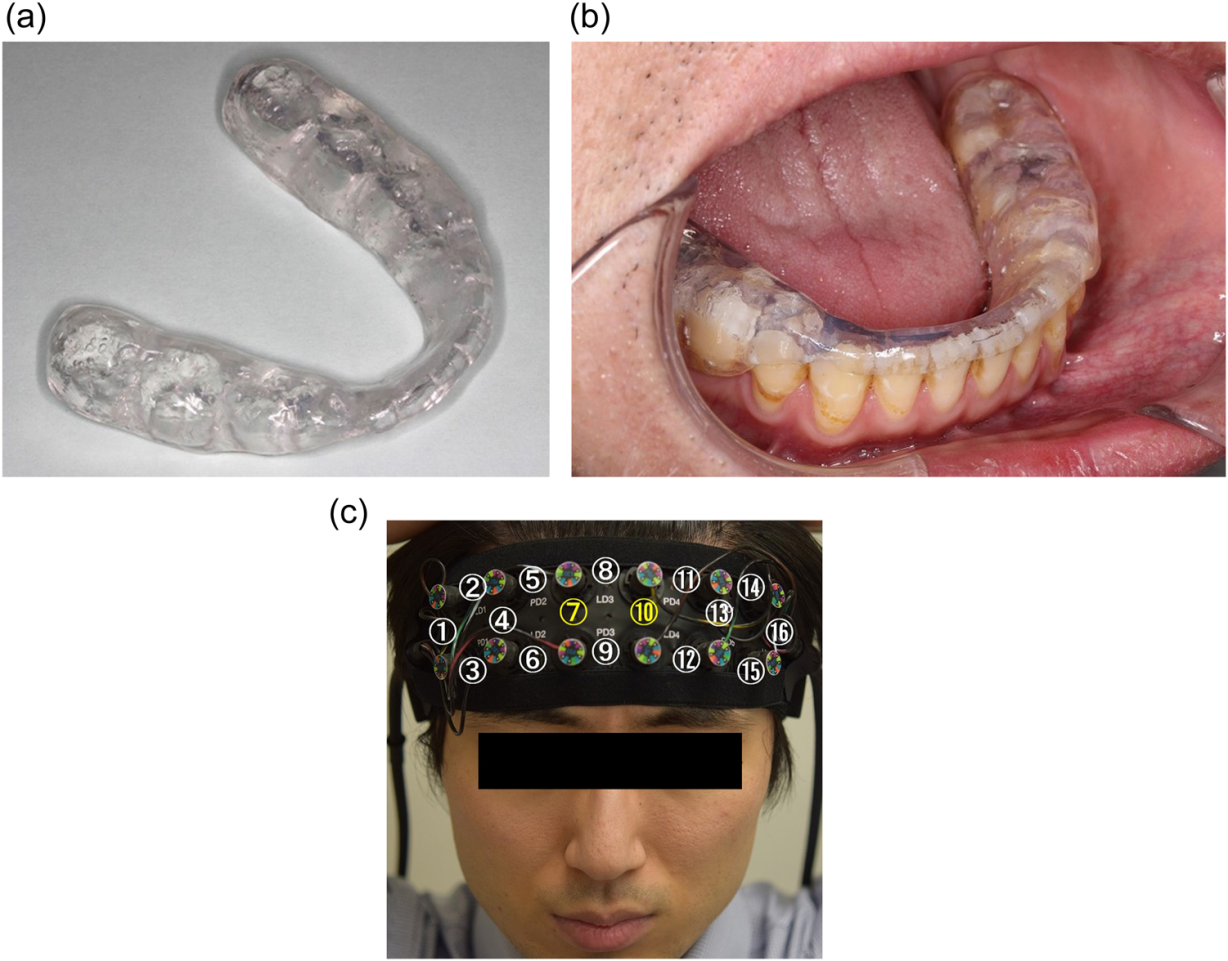
Appearance of the elastic oral appliance. (a) Elastic oral appliance, (b) the elastic oral appliance in the mouth, (c) position of near‐infrared spectroscopy (NIRS) probes. NIRS sensors set on the head. Numbers 1–16 represent the regions covered by channels #1–#16, respectively, and channels #7 and #10 were included in the analysis.

The elastic oral appliance used in this study is shown in Figure 1. The most comfortable material was selected from several materials used in our preliminary experiment. A heat‐curing soft resin (palate resin soft; GC Corp., Tokyo, Japan) that was adjusted to a powder‐to‐liquid ratio of 1:1 was selected. After examining the hardness of various appliances in the preliminary experiment, it was set to 1.89 ± 0.06 N/mm^2^ to minimize discomfort during chewing.

For preparation, first, the impressions of the maxillary and mandibular dentitions of the participants were obtained using an alginate impression material (Algiace‐Z; Dentsply Shirona K.K., Tokyo, Japan). Maxillary and mandibular dental casts were constructed with a type 4 dental stone (New Fujirock; GC Corp., Tokyo, Japan) and were mounted on an average‐value articulator (Handy 2A; Shofu Inc., Kyoto, Japan). Then, a wax pattern was established using paraffin wax (Shofu Inc., Kyoto, Japan) on the mandibular cast to adjust the thickness of the first molar region to 2.5 mm. The teeth surface of the mandibular cast was entirely covered. The cast with the wax pattern was then placed in a flask with dental plaster (Shimomura Gypsum, Saitama, Japan). After the wax was washed out, the heat‐cured soft resin was packed in the flask and polymerized through immersion in water (70°C) for 1 h, followed by immersion in boiling water (100°C) for 1 h. Occlusal contacts on all molars in the habitual occlusal position and on the working and balancing sides in lateral movements were provided. The appliance was adjusted in the mouth before measurement. Previous studies have shown that patients can adapt to an occlusal elevation of 3–5 mm in the dentition with prosthetics and an occlusal elevation of 3.3 mm in the edentulous jaw with little to no difficulty (Moreno‐Hay & Okeson, 2015). For this reason, it was considered that if increasing the vertical dimension was within the interocclusal clearance, the effects on brain activity were minimal.

### 2.3 Experimental design

To compare the influence of chewing on the frontal lobe, two chewing conditions were set: elastic oral appliance chewing and gum chewing. To observe the effect of both chewing conditions on frontal lobe activities, brain mapping and frontal lobe function tests were conducted. A scent‐free tasteless chewing gum (hard type, 1.0 g; Lotte, Tokyo, Japan) was used. The washout duration between each chewing was 5 min. The participants were instructed to chew the elastic oral appliance or gum once per second. At the start of the experiment, the gum was placed in the mouth, and it was not changed between the first and second chewing periods.

A well‐trained dentist performed the assessment in a quiet room with white walls and a temperature controlled at 25°C ± 2°C. The participants were instructed to sit on a chair and position their heads in a manner that the Frankfort plane was parallel to the floor and the mark on the wall was at eye level during chewing.

### 2.4 Measurement of frontal lobe activities

The hemodynamic signals in the brain were measured to investigate its activity. Changes in the frontal lobe were measured using the brain mapping method with 16‐channel near‐infrared spectroscopy (NIRS) (OEG‐16; Spectratech, Tokyo, Japan) according to a previous study (Takeda et al., 2017). The sampling frequency and interval of NIRS were 0.76 Hz and 0.65 s, respectively. The sensor was fixed to the forehead to prevent covering the eyebrows. Channels #7 and #10 were used to analyze separately the changes in blood oxygenation because the noise generated by the temporal muscle during chewing is not likely to affect these channels (Figure 1c). The average values obtained on each channel were used. The values obtained in channels #7 and #10 were used as the representative values for the right and left frontal lobes, respectively.

**Figure 2:**
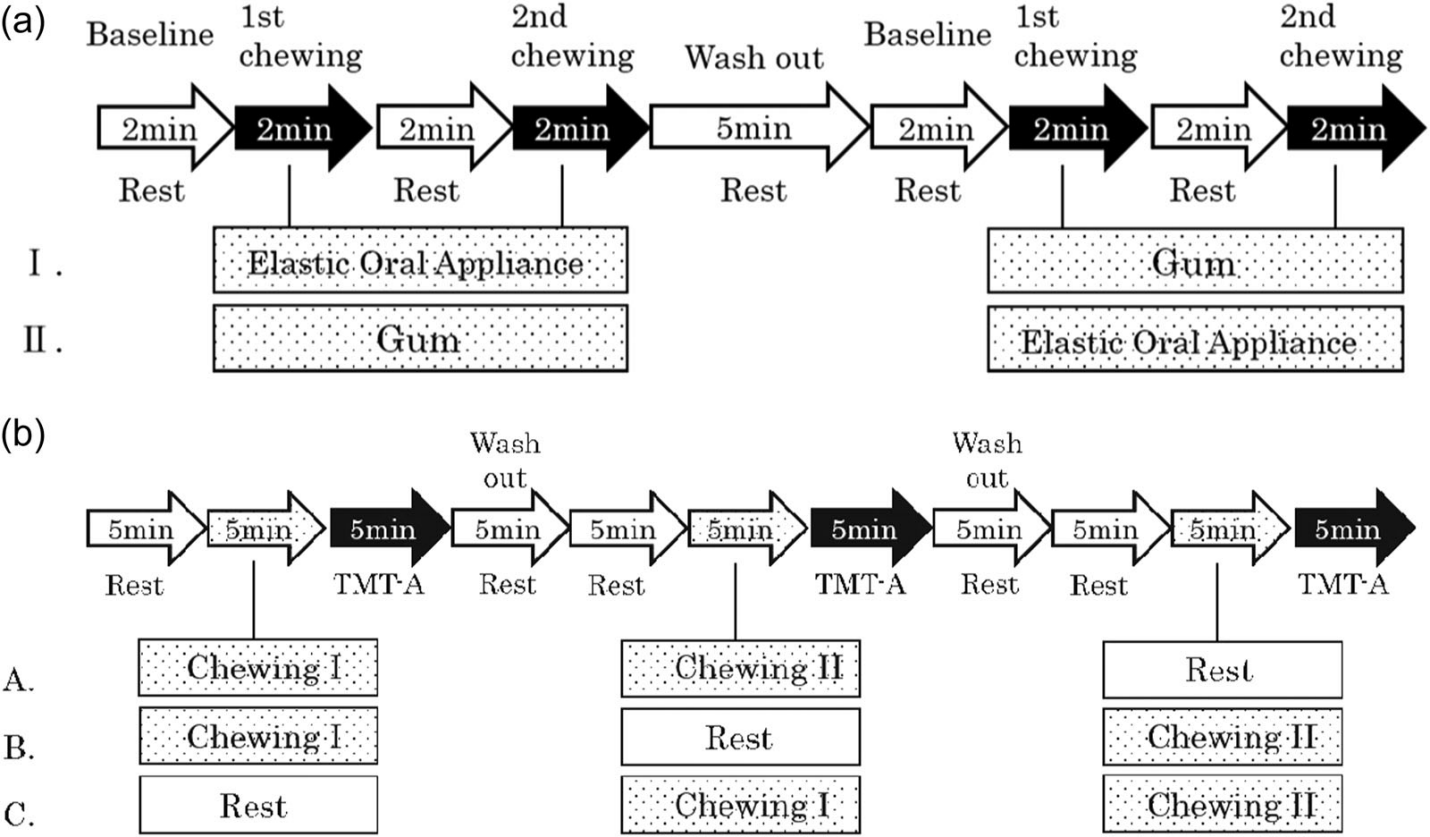
(a) Protocol of the crossover study on near‐infrared spectroscopy (NIRS). This was a prospective crossover study. Experiments were performed in the order presented in I or II. “Elastic oral appliance” and “Gum” represent chewing the elastic oral appliance and chewing gum, respectively. (b) Protocol of the crossover study on Trail Making Test Part A (TMT‐A). This was a prospective crossover study. Experiments were performed in the order presented in A, B, or C. Chewing I and chewing II represent chewing the elastic oral appliance or chewing gum, respectively.

For measurements, changes in blood oxygenation of the frontal lobe were measured in the resting state for 2 min and were considered baseline values. Then, changes in blood oxygenation within the first 2 min of the chewing period and subsequently within the second 2 min were evaluated. A crossover design was utilized for elastic oral appliance and gum chewing (Figure 2a).

### 2.5 Measurement of frontal lobe functions

Frontal lobe functions were measured using the Trail Making Test Part A (TMT‐A). Numbers 1–25 were randomly displayed on a screen, and the participants were asked to select these numbers in ascending order. When the participants finished selecting all numbers accurately, another set of numbers was displayed, and the same process was repeated for 5 min. Then, the mean time for selecting 25 numbers was used as the measured TMT‐A value. A crossover design was utilized for the resting state (control), elastic oral appliance chewing, and gum chewing conditions (Figure 2b). Before measurements, the participants were accustomed to the TMT‐A method.

### 2.6 Statistical analysis

Changes in blood oxygenation in the frontal lobe at baseline and each chewing period were compared using Friedman's test, followed by the Bonferroni test. The TMT‐A values obtained after the resting state and each chewing period were also compared using Friedman's test, followed by the Bonferroni test. A *p*‐value < .05 was considered statistically significant. Statistical analysis was performed using IBM SPSS Statistics for Windows version 24 (IBM Corp., Armonk, NY, USA).

## 3 RESULTS

**Figure 3:**
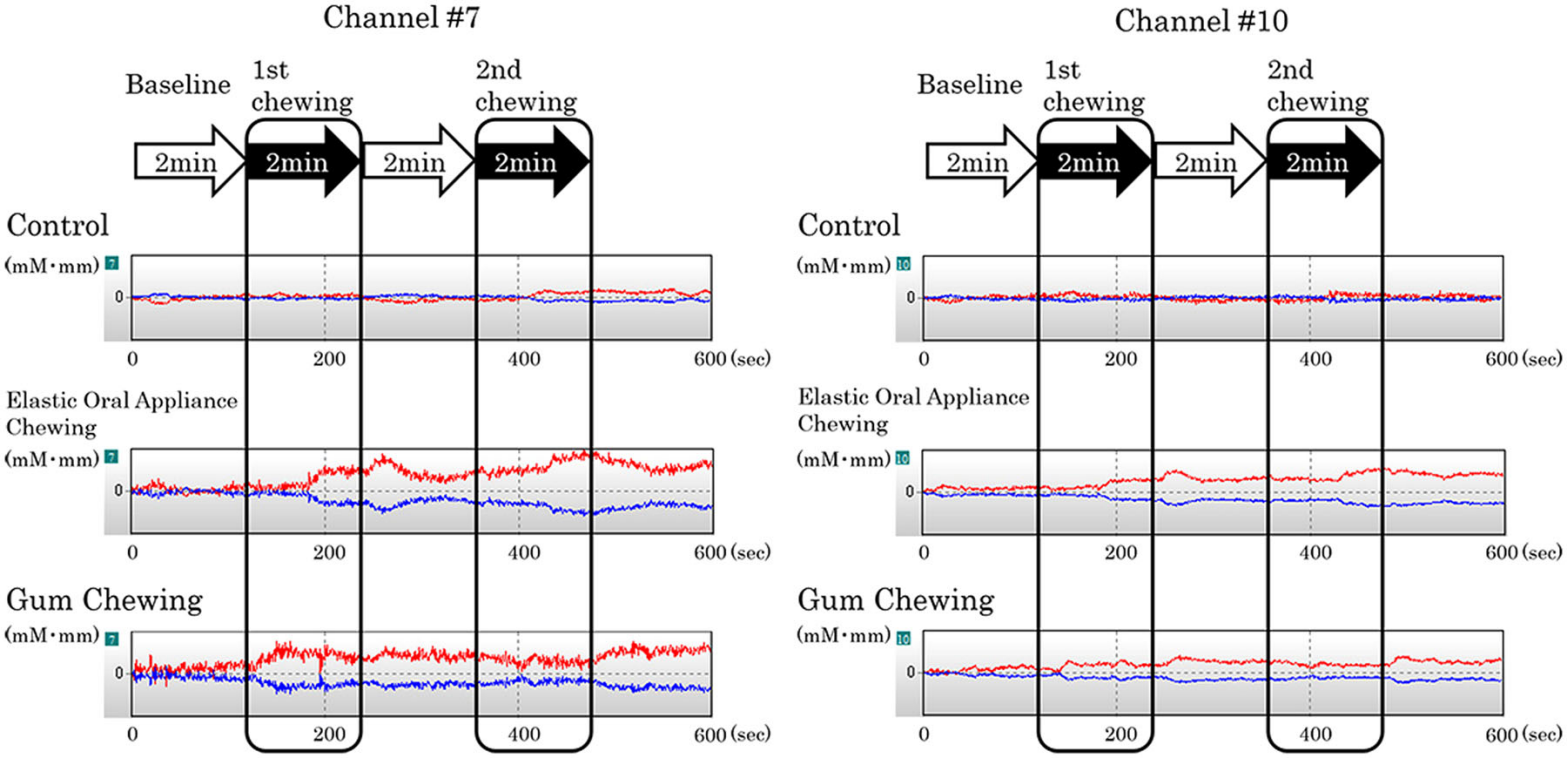
Examples of changes in the blood oxygenation waveforms measured using near‐infrared spectroscopy (NIRS). The waveforms are shown from the top, control, chewing the elastic oral appliance, and chewing gum. Red line: Δoxy‐Hb concentration, Blue line: Δdeoxy‐Hb concentration.

**Figure 4:**
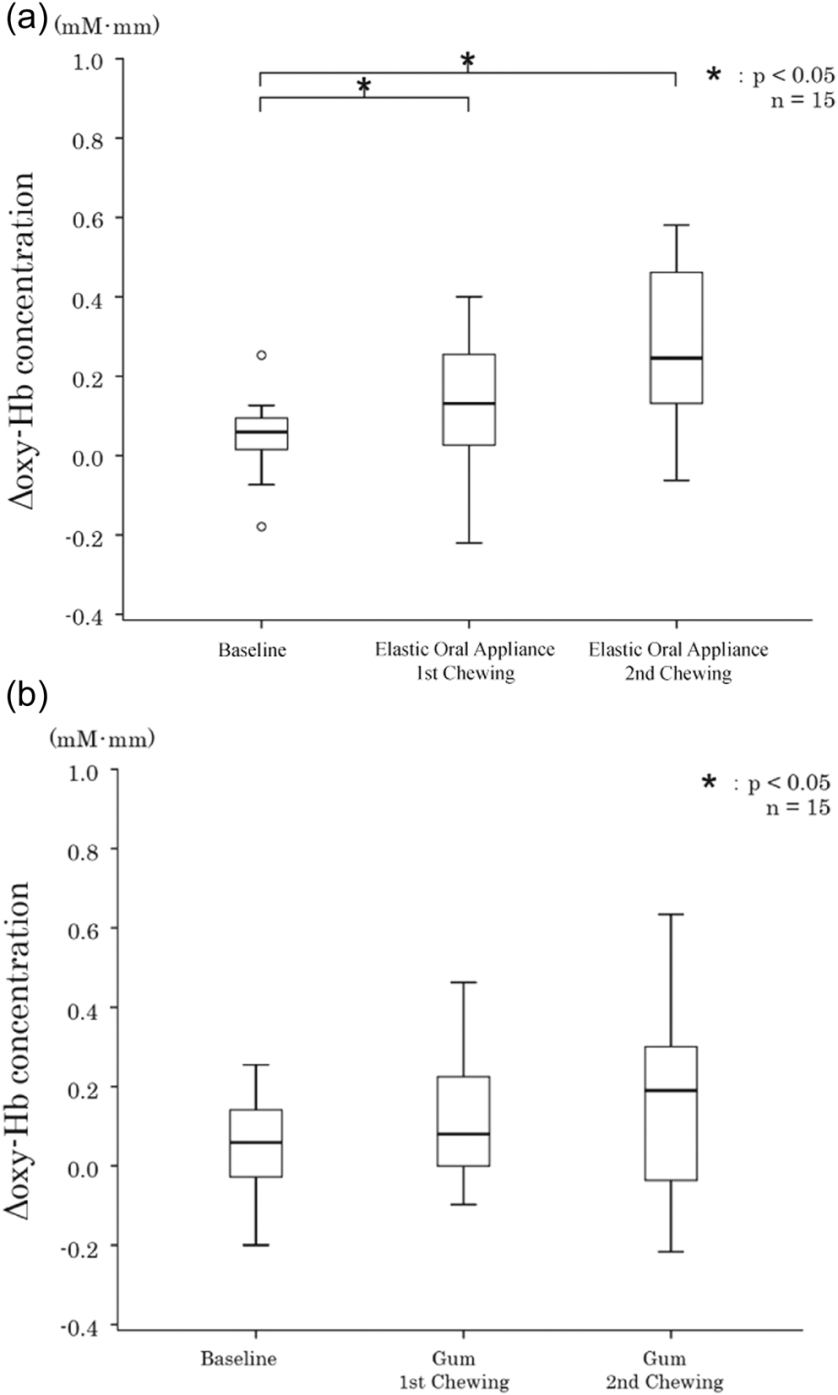
Results of changes in blood oxygenation in the right frontal lobe. (a) Chewing the elastic oral appliance, (b) chewing gum.

Changes in blood oxygenation waveform, which were measured using NIRS, are shown in Figure 3. During the second chewing period, elastic oral appliance and gum chewing increased blood oxygenation on channel #10. In the same period, elastic oral appliance chewing increased blood oxygenation on channel #7. When chewing the elastic oral appliance, the median (interquartile range) values of blood oxygenation on channel #7 at baseline, first chewing period, and second chewing period were 0.06 (0.00–0.10), 0.13 (0.02–0.26), and 0.25 (0.10–0.54) mM • mm, respectively. Significant differences were found between the baseline and the first chewing period (*p* = .032) and between the baseline and the second chewing period (*p* < .001) (Figure 4a).

When chewing the elastic oral appliance, the median (interquartile range) values of blood oxygenation on channel #10 at baseline, first chewing period, and second chewing period were 0.08 (0.02–0.16), 0.19 (0.14–0.32), and 0.28 (0.24–0.52) mM • mm, respectively. Furthermore, a significant difference was observed between the baseline and the first chewing period (*p* = .204) and between the baseline and the second chewing period (*p* < .001) (Figure 4b).

**Figure 5:**
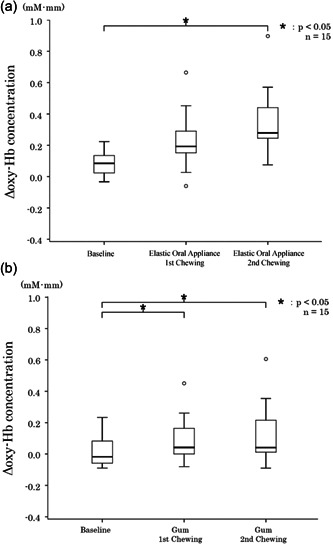
Results of changes in blood oxygenation in the left frontal lobe. (a) Chewing the elastic oral appliance, (b) chewing gum.

With gum chewing, the median (interquartile range) values of blood oxygenation on channel #7 at baseline, first chewing period, and second chewing period were 0.06 (−0.03–0.16), 0.08 (−0.01–0.24), and 0.19 (−0.04–0.30) mM • mm, respectively, and no significant difference was found in the values using the Friedman test (*p* = .085) (Figure 5a).

During gum chewing, the median (interquartile range) values of blood oxygenation on channel #10 at baseline, first chewing period, and second chewing period were −0.02 (−0.06–0.14), 0.04 (−0.01–0.04), and 0.04 (0.00–0.26) mM • mm, respectively. Furthermore, a significant difference was observed in the values between the baseline and the first chewing period (*p* = .032) and between the baseline and the second chewing period (*p* = .01) (Figure 5b).

**Figure 6:**
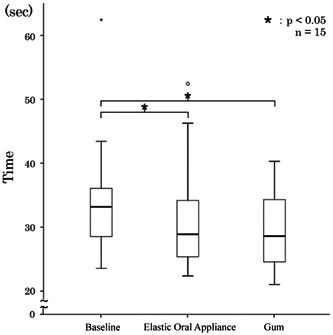
Results of the Trail Making Test‐A

The TMT‐A test results after resting, elastic oral appliance chewing, and gum chewing were 33.19 (28.76–35.82), 28.89 (25.54–33.56), and 28.60 (24.89–33.99) s, respectively. Significant differences were found in the test results between the resting state and elastic oral appliance chewing (*p* = .04) and between the resting state and gum chewing (*p* = .002) (Figure 6).

## 4 DISCUSSION

Since the brain mapping method can measure frontal lobe activities, positron emission tomography (PET) has been widely used as it quantifies local cerebral changes in blood oxygenation and metabolism. Moreover, it measures frontal lobe activities (Gordh et al., 2010); however, PET exposes the participants to radiation. By contrast, NIRS can quantify local frontal lobe activities without such exposure. The maximum measurable depth of NIRS at a sensor interval of 3.5 cm is 4 cm, (Okada et al., 1993), and this spatial resolution is lower than that of PET. The level of frontal lobe activities evaluated using NIRS is associated with that assessed using PET (Hasegawa et al., 2013; Hock et al., 1997; Villringer et al., 1997).

This study was conducted to investigate the effect of chewing on brain activity. Thus, a high spatial resolution analysis was unnecessary. Accordingly, NIRS was utilized, and the average changes in blood oxygenation in the frontal lobe while chewing were assessed.

As frontal lobe activities cannot be measured using only one method, TMT‐A was used in addition to NIRS. A previous study revealed a correlation between the volume of the active brain region, including the frontal lobe, evaluated via functional magnetic resonance imaging and TMT‐A‐assessed value (Zakzanis et al., 2005).

Changes in blood oxygenation in the frontal lobe increased when chewing the elastic oral appliance, which is similar to the frontal lobe activities observed during gum chewing. Thus, the elastic oral appliance chewing exercise improved frontal lobe activities. Somatosensory stimulation by mastication and proprioceptive stimulation from the masticatory muscles reach the primary somatosensory area via the thalamus (Ono et al., 2010). The activity of the primary somatosensory area stimulates the frontal lobe and increases PFC activity (Onozuka et al., 2002).

In the second chewing period, the elastic oral appliance chewing exercise improved the activity on both sides of the frontal lobe. By contrast, gum chewing improved only the frontal lobe activities on the left side (channel #10). This result indicates that the elastic oral appliance stimulated the teeth on both sides during chewing, whereas gum chewing stimulated only one side. However, this study examined the effects of chewing with elastic oral appliances on frontal lobe activities. Since both areas are in the frontal lobe, it was not necessary to compare the activities on the left and right sides.

The tactile sensation of the teeth occurs through the periodontal ligament mechanoreceptor, (Dong et al., 1993), and frontal lobe activities increased with vibrotactile sensation from the periodontal ligament (Ettlin et al., 2004; Trulsson et al., 2010). Furthermore, chewing exercises increase the activities of the sensorimotor area cortex, supplementary motor area, thalamus, and frontal cortex (Kimoto et al., 2011). Based on these findings, oral stimulation by chewing an elastic oral appliance may also have occurred via the oral mucosa, periosteum, and periodontal ligament, thereby increasing frontal lobe activities. During the gum chewing exercise, the gum comes in contact with a part of the dentition. Meanwhile, during the elastic oral appliance chewing exercise, the elastic oral appliance comes in contact with more teeth and a large area, which may increase the oral somatosensory stimuli.

Studies on the influence of external stimuli on the brain have revealed that brain activities were improved in both sexes (Gao et al., 2018; Okada et al., 1993). In another study, the reaction was unilateral in men, whereas it was bilateral in women, (Okada et al., 1993), demonstrating a slight sex difference in the reactivity. As the goal of this study was to observe chewing‐induced improvements in frontal lobe activities, limiting the sex of the participants to men or women was considered important.

This study had some limitations. The brain responds to external stimuli more clearly and with lesser variation in young than in older individuals. Hence, only young participants were included, and the effect of the elastic oral appliance chewing exercise on frontal lobe activities among older and edentulous individuals was not investigated. Furthermore, conduction pathways of somatosensory perception by external stimuli are widely known and should not change with age. Therefore, although the degree of impact may be different in older people, results in this study will unlikely be reversed in older people. However, the effects of the elastic oral appliance chewing exercise on brain activities in older people may be different from those in younger participants; thus, further research is needed. The somatosensory stimulation of regions other than the periodontal ligament affected frontal lobe activities. Based on the finding about the effect of somatosensory stimulation of regions other than the periodontal ligament on frontal lobe activities, (Kimoto et al., 2011) the elastic oral appliance chewing exercise may increase frontal lobe activities in individuals wearing complete dentures who have no periodontal ligament. Moreover, the elastic oral appliance chewing exercise might prevent further decline in cognitive functions among older individuals. However, further studies should assess the effect of the elastic oral appliance chewing exercise on cognitive functions among older individuals. Furthermore, the elastic modulus of the appliance was 1.89 ± 0.06 N/mm^2^. However, gum hardness was measured as viscosity. Thus, the hardness of the elastic oral appliance and the gum cannot be compared.

An elastic oral appliance or gum chewing improved blood oxygenation in the frontal lobe, indicating that the elastic oral appliance chewing exercise could increase frontal lobe activities. This finding is consistent with the TMT‐A results. In addition, the elastic oral appliance chewing exercise may influence frontal lobe activities through a mechanism similar to gum chewing. Thus, this exercise may increase frontal lobe activities among denture wearers, thereby improving memory, learning ability, (Fukushima‐Nakayama et al., 2017; Jiang et al., 2015), and speed of response to stimulation (Hirano et al., 2013; Sakamoto et al., 2009). In addition, our results suggest that the elastic oral appliance chewing exercise may increase frontal lobe activities without food or gum.

## 5 CONCLUSIONS

Elastic oral appliance chewing improved the activities and functions of the frontal lobe. The results suggest that elastic oral appliance chewing improves frontal lobe activities to a level similar to those obtained with gum chewing.

## AUTHOR CONTRIBUTIONS

*Conception and design*: Yuu Yamamoto, Masahiro Ryu, Takayuki Ueda, and Yoshinori Sasaki, Kaoru Sakurai. *Acquisition, analysis, and interpretation of data*: Yuu Yamamoto. *Drafting the manuscript*: Yuu Yamamoto and Masahiro Ryu. *Revising the manuscript critically for important intellectual content*: Takayuki Ueda, Yoshinori Sasaki, and Kaoru Sakurai.

## CONFLICT OF INTEREST

The authors declare no conflicts of interest.

## Data Availability

The data that support the findings of this study are available from the corresponding author upon reasonable request.
